# Curcumin and Its Derivatives as Potential Antimalarial and Anti-Inflammatory Agents: A Review on Structure–Activity Relationship and Mechanism of Action

**DOI:** 10.3390/ph16040609

**Published:** 2023-04-18

**Authors:** Siti Nur Hidayah Jamil, Amatul Hamizah Ali, Shevin Rizal Feroz, Su Datt Lam, Hani Kartini Agustar, Mohd Ridzuan Mohd Abd Razak, Jalifah Latip

**Affiliations:** 1 Department of Chemical Sciences, Faculty of Science and Technology, Universiti Kebangsaan Malaysia (UKM), Bangi 43600, Selangor, Malaysia; 2 Department of Biological Sciences and Biotechnology, Faculty of Science and Technology, Universiti Kebangsaan Malaysia (UKM), Bangi 43600, Selangor, Malaysia; 3 Department of Applied Physics, Faculty of Science and Technology, Universiti Kebangsaan Malaysia (UKM), Bangi 43600, Selangor, Malaysia; 4 Department of Earth Sciences and Environment, Faculty of Science and Technology, Universiti Kebangsaan Malaysia (UKM), Bangi 43600, Selangor, Malaysia; 5 Herbal Medicine Research Centre, Institute for Medical Research, National Institute of Health (NIH) Complex, Ministry of Health Malaysia, Shah Alam 40170, Selangor, Malaysia

**Keywords:** curcumin derivatives, antimalaria, anti-inflammatory, structure–activity relationship, molecular targets

## Abstract

Curcumin, one of the major ingredients of turmeric (*Curcuma longa*), has been widely reported for its diverse bioactivities, including against malaria and inflammatory-related diseases. However, curcumin’s low bioavailability limits its potential as an antimalarial and anti-inflammatory agent. Therefore, research on the design and synthesis of novel curcumin derivatives is being actively pursued to improve the pharmacokinetic profile and efficacy of curcumin. This review discusses the antimalarial and anti-inflammatory activities and the structure–activity relationship (SAR), as well as the mechanisms of action of curcumin and its derivatives in malarial treatment. This review provides information on the identification of the methoxy phenyl group responsible for the antimalarial activity and the potential sites and functional groups of curcumin for structural modification to improve its antimalarial and anti-inflammatory actions, as well as potential molecular targets of curcumin derivatives in the context of malaria and inflammation.

## 1. Introduction

**Figure 1:**
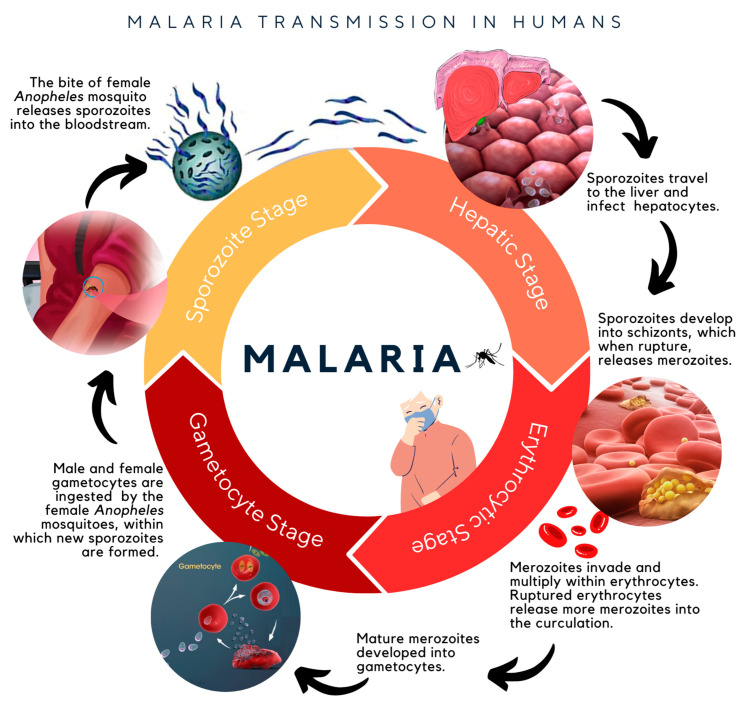
The life cycle of the *Plasmodium* parasite, beginning from the infection of sporozoites released by the bite of a female *Anopheles* mosquito into the circulatory system of humans [1,4].

Malaria is a highly life-threatening infectious disease caused by the transmission of five *Plasmodium* parasites infecting the human body, which are *P. falciparum*, *P. vivax*, *P. ovale*, *P. malariae*, and *P. knowlesi*. *P. falciparum*, identified as being the most common and feared species infecting humans, is transmitted into the circulatory system through the infective bite of female *Anopheles* mosquitoes. This triggers the development of sporozoites, which can rapidly infect liver cells within 30 min after a bite (Figure 1). After approximately five days, the sporozoites mature into merozoites, and upon release from the cell, will begin to invade erythrocytes. The merozoites then multiply within the erythrocytes, leading to the release of more invasive merozoites to infect other erythrocytes. At this point, malarial-associated symptoms, namely, fever, headaches, body aches, general malaise, and rigors, will become apparent [1]. At the same time, mature merozoites will continue their development into gametocytes (sexual-stage parasites), which will subsequently form new sporozoites within female *Anopheles* mosquitoes to complete the *Plasmodium* life cycle [2,3]. 

**Figure 2:**
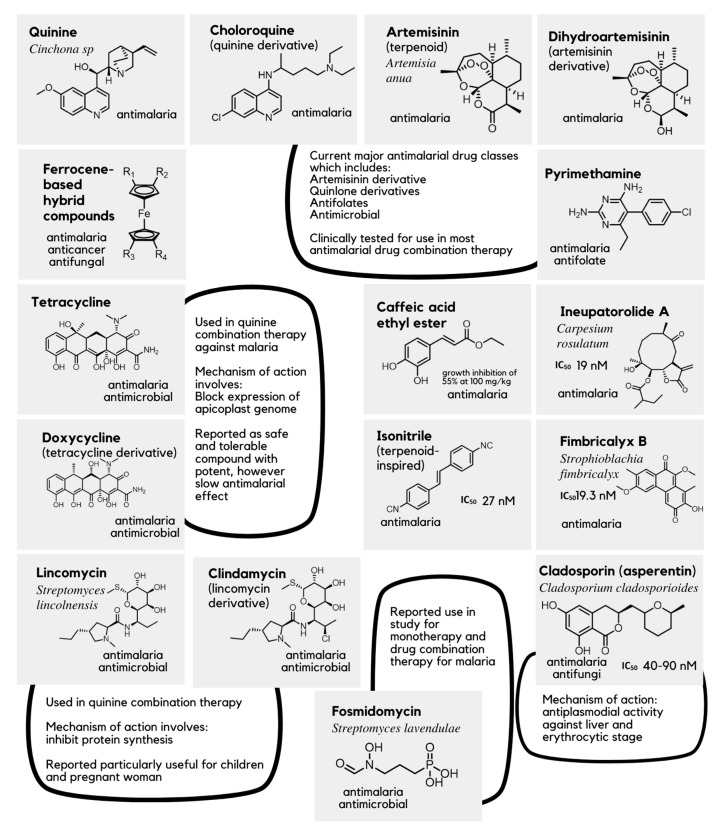
Currently available drugs and biologically active compounds used in the research for treatment of malaria. [2,3,8,9].

Recent statistical data from the World Health Organization (WHO) indicate that 1.7 billion cases of malaria have been recorded worldwide, which led to 10.6 million deaths within the previous 20 years (2000–2020) [5]. *Plasmodium* infection and the consequent inflammation can lead to advanced and fatal malaria if left untreated, including cerebral and severe malaria, and other complications such as cardiovascular diseases. With severe and cerebral malaria being leading causes of mortality, many studies are currently focused on developing effective antimalarial and anti-inflammatory treatments. In addition, therapeutic efficacy studies (TESs) have shown increasing drug resistance in malaria, where currently available antimalarial drugs such as artemisinin and chloroquine are no longer effective against malaria infection. In line with the primary goal of the Global Fund Strategy 2023–2028, sourcing and developing novel and potent inhibitors to combat drug resistance in malaria treatment is now a necessity, hence, the growing list of bioactive compounds, which are proven for their antimalarial activities (Figure 2) [2,3,5,6,7].

**Figure 3:**
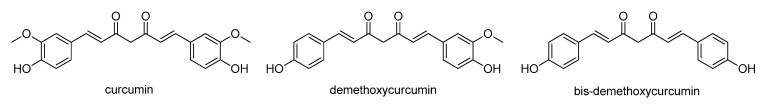
The chemical structures of curcumin and two other curcuminoids naturally present in commercially available curcumin.

Curcumin, which has been proven to be a promising candidate as an antimalarial and anti-inflammatory agent, is a natural polyphenolic compound, originating from the rhizomatous perennial plant, turmeric [10,11]. Curcumin was first named and reported by Vogel and Pelletier as a compound isolated from *Curcuma longa* rhizomes and was identified as an “orange-yellow substance” [12]. In commercially available curcumin, curcumin is present as a mixture of three curcuminoids, namely, curcumin (77%), bisdemethoxycurcumin (3%), and demethoxycurcumin (17%) [13] (Figure 3). Due to its high abundance among curcuminoids, curcumin is more accessible for research and drug development compared to other naturally sourced bioactive compounds that are present in low concentrations in the source plants [2].

Curcumin is known for its diverse applications worldwide. Aside from its use as a therapeutic remedy such as in traditional medicine and as an antiseptic agent, it is a popular cooking ingredient in many Asian countries. Further, it is applied as a coloring agent in manufacturing, food and beverage, and cosmetic industries [14]. Curcumin, which has been proven to show no toxicity even at high doses [15,16], has been approved as a “Generally Recognized as Safe” (GRAS) compound by the US Food and Drug Administration (FDA) [13]. However, the low bioavailability of curcumin limits its clinical applications and presents challenges in developing curcumin into a potent antimalarial drug candidate due to its poor oral absorbability, low aqueous solubility, and rapid metabolism in the body [17]. 

This review identifies curcumin derivatives with reported antimalarial and anti-inflammatory properties, evaluates the key functional groups/sites responsible for the antimalarial and anti-inflammatory activities, and discusses the mechanisms of action of these compounds that are associated with their biological effects. Several approaches to improve the activity of curcumin, including through drug combination, adjuvant application, curcumin nanoencapsulation, and structural modifications, have been previously suggested [18]. A specific approach focusing on the structural modification of the parent curcumin to enhance its bioactivity as an antimalarial and anti-inflammatory agent based on structure–activity relationship (SAR) analysis will also be expounded in this review. In addition, the absorption, distribution, metabolism, and excretion (ADME) properties of the curcumin derivatives will also be reviewed to evaluate their pharmacological profiles. Thus, this work is expected to be helpful in understanding the potential enhancement of curcumin bioactivity in malaria and inflammatory-related diseases, as well as in elucidating a detailed molecular-level mechanism of action associated with malarial infection.

## 2. Antimalarial and Anti-Inflammatory Activities of Curcumin and Its Structural Modifications

**Figure 4:**
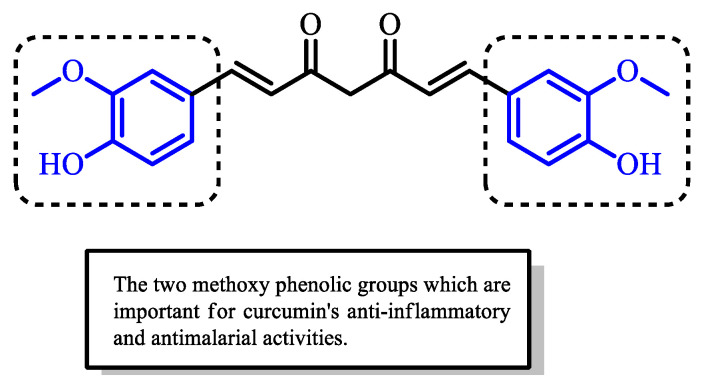
Chemical structure of curcumin with its two methoxy phenolic groups.

**Figure 5:**
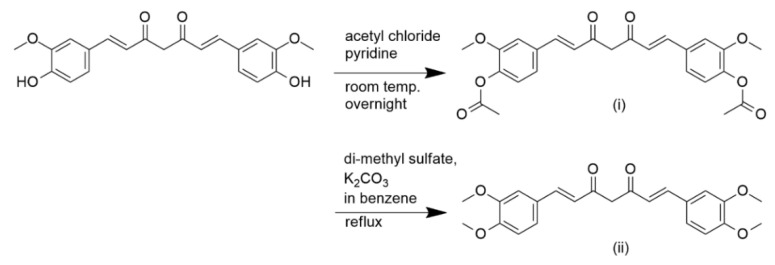
The reaction scheme for the synthesis of di-O-acetyl and di-methoxycurcumin involving substitution of the phenolic OH group [37].

Curcumin has been reported to possess multiple therapeutic effects, including anti-inflammatory [19,20,21,22], anti-plasmodial [16,23,24,25], antifungal [16,26,27], antibacterial [28,29,30], antioxidant [31,32,33], and antitumor [16,34,35,36] activities. An understanding of its chemical structure has led to the identification of the sites responsible for its bioactivity, which are primarily its two methoxy phenolic groups (Figure 4). The hydroxy group attached at the para position of the aromatic phenyl group contributes toward the stability of curcumin, as the electrons from the hydroxy group are delocalized into the aromatic ring. Dohutia et al. investigated the effect of substituting the hydroxy group of curcumin with O-acetyl and methoxy groups on its antimalarial activity (Figure 5). The IC_50_ values from the in vitro study showed that substitution with an O-acetyl group (IC_50_ = 2.34 µM) exhibited similar potency as curcumin (IC_50_ = 3.25 µM); however, the potency decreased markedly (IC_50_ = 7.86 µM) when substituting with a methoxy group [37]. This was thought to be due to the biodegradability of the acetyl group through ester bond cleavage by esterases, which regenerates the parent curcumin structure, in contrast to the bond attached to an alkyl group, which is not readily cleaved [38]. Hence, this finding revealed the importance of the unsubstituted phenol group since the modification of the site led to the loss of curcumin’s antimalarial activity [38,39].

The need to develop potent inhibitors of proteins involved in the pathogenesis of malaria pathways is currently at a crisis point due to the increasing resistance of *Plasmodium* parasites against present antimalarial drugs and the lack of lead compounds for new antimalarial and anti-inflammatory agents. Thus, one approach that is actively implemented for malaria treatment is drug combination therapy [5,40,41,42,43]. Such an approach for the treatment of various diseases has long been applied, for example, to reduce drug resistance for tuberculosis in the 1960s [36]. According to the WHO, current front-line medications designed to mitigate the resistance of *Plasmodium* parasites against present antimalarial drugs are often based on artemisinin combination therapy (ACT), involving the combination of artemether–lumefantrine and amodiaquine (AL-AQ) [5]. Curcumin has also been used as a component in combination therapy against malaria. For instance, Tjahjani et al. showed that curcumin produced a synergistic antimalarial effect when administered together with dihydroartemisinin [44]. Furthermore, the combination of curcumin and piperine, an active compound found in black pepper, has proven to significantly enhance the bioavailability of curcumin by 2000% compared to curcumin alone [41]. 

Although drug combination therapy is proven for its efficacy, a potential drawback to its application are side effects, which are more likely compared to single-drug therapy [42]. Therefore, drug discovery by way of structural modification of existing bioactive compounds remains an important approach to combat drug resistance, along with drug combination therapy. The bioactivity of curcumin against the infected host and parasite target proteins is highly dependent on its reactivity [45,46]. However, the chemical structure of curcumin limits its bioavailability and stability at physiological pH and, thus, its efficacy as an antimalarial and anti-inflammatory agent [15,40]. Factors such as poor absorption, rapid metabolism, and rapid elimination contribute to the low bioavailability of curcumin in the body [15,47,48,49]. Therefore, deriving compounds with better pharmacokinetic properties than the parent curcumin is important in developing compounds with enhanced anti-inflammatory and antimalarial activities. 

**Figure 6:**
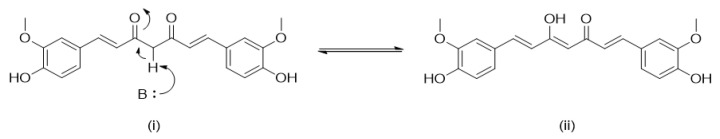
The equilibrium between the (**i**) keto and (**ii**) enolate forms of curcumin due to the reactivity of the methylene α-hydrogen, which is susceptible to nucleophilic attack, leading to the low stability of curcumin [45,53,54].

**Figure 7:**
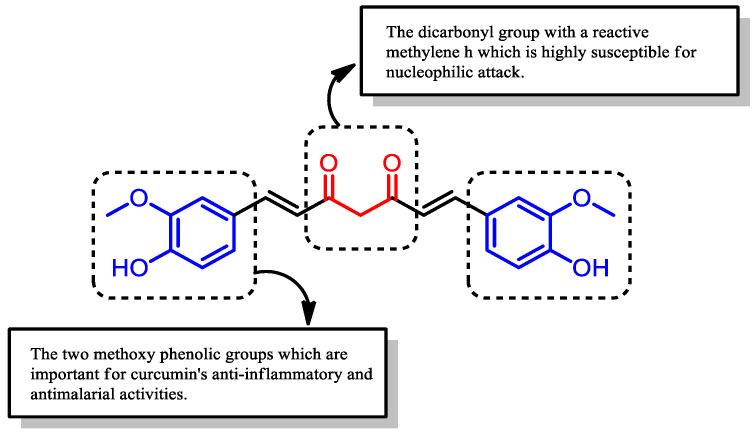
Specific sites and functional groups of curcumin, which can be targeted for structural modifications.

**Figure 8:**
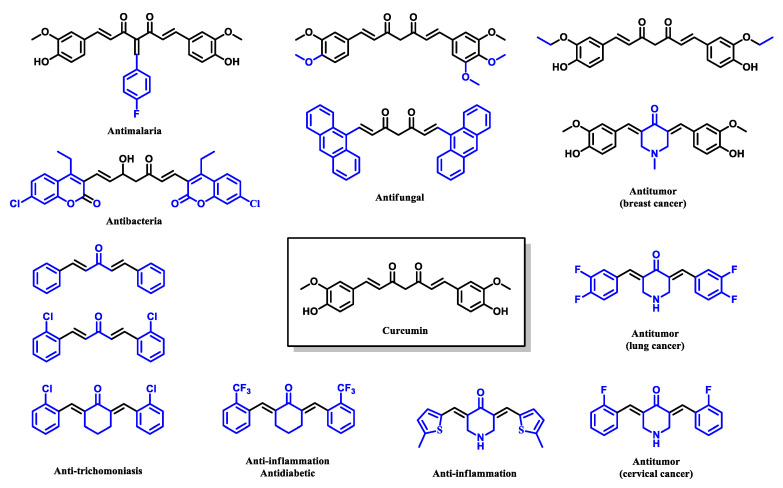
Examples of curcumin derivatives with a wide range of therapeutic properties [9,38,50,55,56,57,58,59,60,61].

The instability and low bioavailability of curcumin are mainly due to the presence of the dicarbonyl group with a highly acidic α-H, which can easily resonate between keto and enolate forms, as illustrated in Figure 6 [50]. Previous research has indicated that the extremely reactive β-diketone group appears to be highly unstable and can be quickly degraded. Hence, curcumin derivatives are being actively studied to overcome the pharmacological limitations due to the chemistry of curcumin (Figure 7). Examples of curcumin derivatives exhibiting reasonable and better efficacy against various diseases than the parent curcumin are shown in Figure 8. A common strategy in designing curcumin derivatives with enhanced bioactivity is by lowering rotational flexibility and producing a more rigid orientation by reducing the reactivity of methylene alpha-H of the β-diketone moiety [51,52]. 

Before structural modification of curcumin is performed, the potential enhancement of curcumin bioactivity can be preliminarily suggested through pharmacokinetic analysis. Pharmacokinetic analysis is useful to predict the absorption, distribution, metabolism, and excretion (ADME) profiles of a compound and, thus, can provide supplementary screening data to evaluate the possible effects of a compound upon administration into the body. 

**Figure 9:**
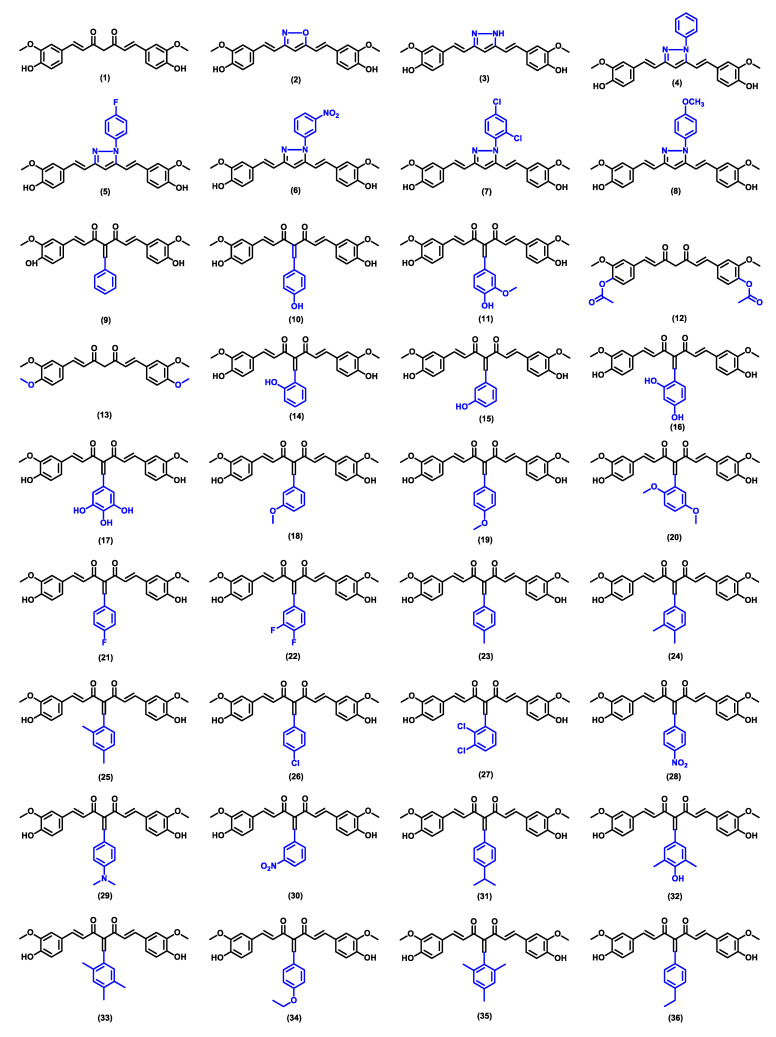

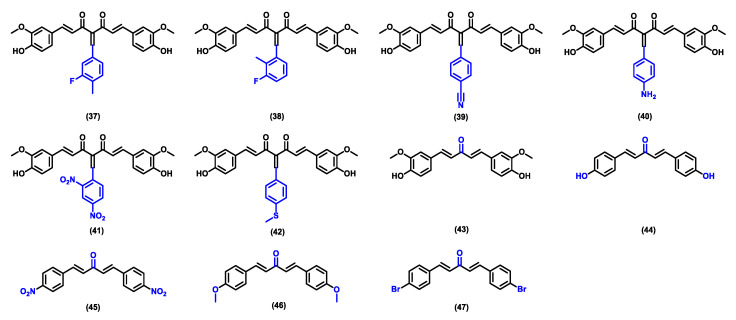
Chemical structures of curcumin derivatives, which have been synthesized and studied for their anti-inflammatory and antimalarial activities [23,47].

The Swiss ADME server was employed to evaluate the potential of curcumin derivatives over the parent curcumin as drug candidates with enhanced antimalarial activity [62]. Table 1 shows the results of the analysis, which includes the predictions of physicochemical properties, drug-likeness according to Lipinski’s Rule of Five, and pharmacokinetic parameters for curcumin and its derivatives, as illustrated in Figure 9. The data also provide an overview of the ability of the compounds to form hydrogen bonds, which can be indicators of favorable interaction with protein active sites. 

The majority of curcumin derivatives illustrated in Figure 9 are predicted to have better solubility, better GI absorbability, and BBB permeability than the parent curcumin. Enhanced interactions with the active site of proteins would theoretically delay the metabolism and elimination of a drug, hence, prolonging its duration of action. This analysis provides information to understand the possible enhancement of bioactivity contributed by structurally modifying the parent curcumin, which will be discussed in more detail in the later parts of this review.

## 3. Structure–Activity Relationship of Curcumin Derivatives as Antimalarial and Anti-Inflammatory Agents

The presence of various functional groups can influence the pharmacodynamic and pharmacokinetic effects of a drug. The unsubstituted methoxy phenol group of curcumin has been identified as crucial for its antimalarial and anti-inflammatory activity. Hence, the structural modification of curcumin is mostly focused on the β-dicarbonyl group and the highly acidic methylene α-hydrogen bridging the dicarbonyl groups.

**Figure 10:**
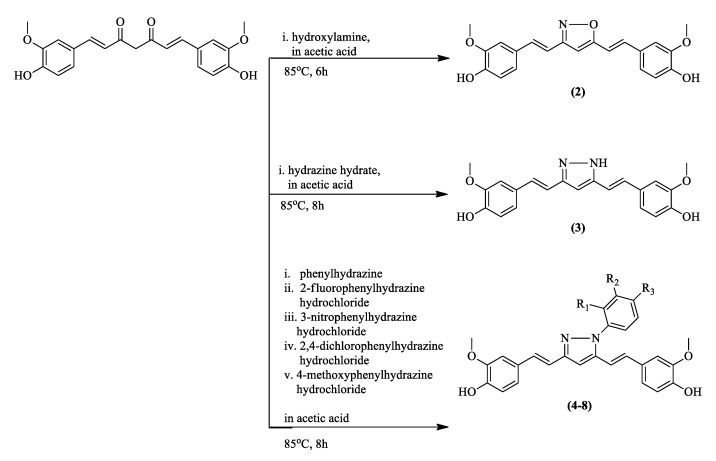
Reaction scheme showing the synthesis of isoxazole and pyrazole derivatives of curcumin.

In a study by Mishra et al., the keto–enol moiety was converted into the more rigid five-membered isoxazole and pyrazole rings, targeting the reactive methylene α-hydrogen [38,63]. The isoxazole and pyrazole derivatives of curcumin were synthesized based on the previously reported reaction schemes, as illustrated in Figure 10 [38,64,65]. The isoxazole and pyrazole derivatives have been reported for their improved anti-inflammatory and antiplasmodial effects, with the latter based on in vitro assays against both chloroquine-sensitive and chloroquine-resistant *P. falciparum* [38] (Table 2).

**Figure 11:**
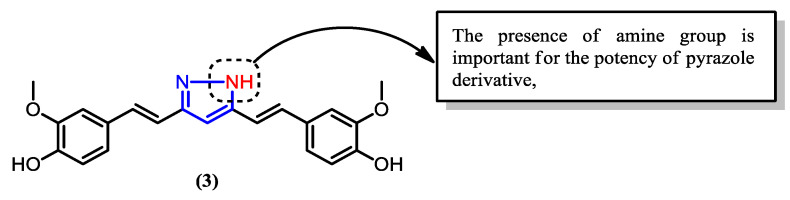
The pyrazole derivative of curcumin has higher efficacy than the isoxazole derivative.

Interestingly, the isoxazole derivative (**2**) exhibited reduced antiplasmodial potency compared to the parent curcumin. Changing the oxygen atom of the isoxazole ring into a nitrogen atom, thus, forming the pyrazole derivative (**3**) (Figure 11), greatly enhanced the activity, with an IC_50_ value of 0.48 μM, compared to 3.25 μM of the parent curcumin. Thus, the presence of nitrogen is vital for the antimalarial activity of the curcumin derivative.

**Figure 12:**
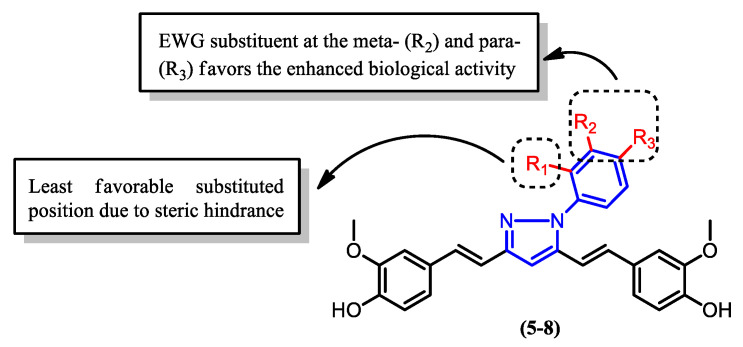
Pyrazole derivatives of curcumin with a phenyl group attached to the nitrogen atom. The presence of different groups, either electron donating (EDG) or electron withdrawing (EWG), on the phenyl group varyingly influences the bioactivity of the compounds.

**Figure 13:**
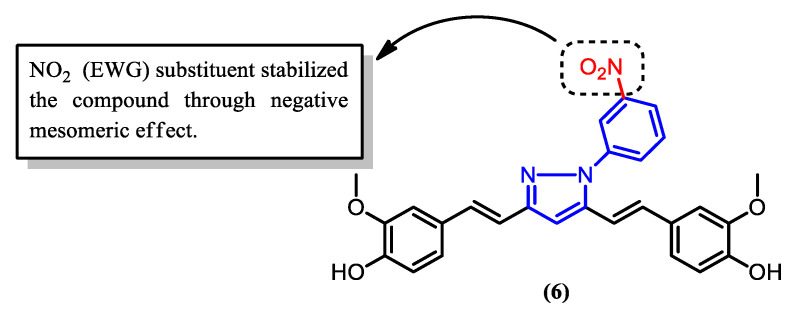
Pyrazole derivative (**6**) with a nitro substituent at the phenyl ring, which contributes to its enhanced bioactivity.

The activity of the pyrazole derivatives (**4**–**8**) upon the addition of a phenyl group to the nitrogen atom was also investigated (Figure 12). The formation of derivatives (**5**), (**6**), and (**7**) revealed that the presence of different functional groups on the phenyl ring greatly affects the potency of the compounds. Derivative (**5**), which contains fluorine as opposed to chlorine, as in derivative (**7**), showed significantly higher activity, as can be seen in Table 2. This can be attributed to the lower steric hindrance caused by fluorine compared to chlorine, leading to a more favorable structural characteristic for interaction with other molecules. In addition, the low IC_50_ value of derivative (**6**), which contains a nitro group attached to the phenyl (Figure 13), indicates that the electron withdrawing group enhances the activity and potency of curcumin derivatives against malaria. This is believed to be due to the negative mesomeric effect, which delocalized and withdrew electrons from the ring, hence stabilizing the compound [66].

Similar to derivatives synthesized by the Knoevenagel reaction (**9**, **10**, **11**, and **14**–**42**), the presence of an electron donating group (**8**) resulted in a negative effect on the antimalarial activity of the derivative, with lower potency compared to the parent curcumin. Therefore, these observations demonstrate that electron-withdrawing substituents are crucial to enhancing the antimalarial activity of curcumin derivatives. 

**Figure 14:**
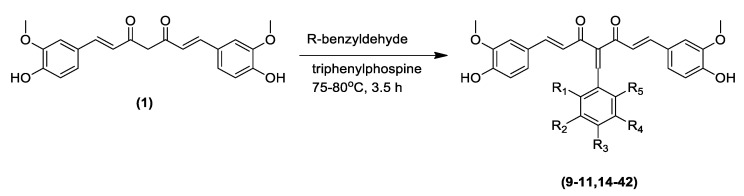
The reaction scheme and the chemical structures of derivatives (**14**) and (**15**), as synthesized by Knoevenagel condensation [37,38,70].

**Figure 15:**
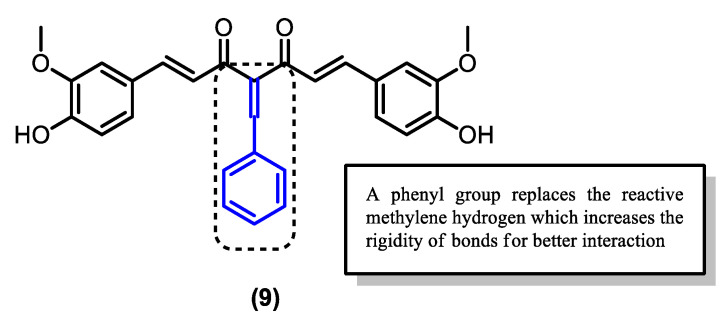
Curcumin derivative whose active methylene group is substituted by Knoevenagel condensation.

Apart from the pyrazole derivatives, a series of Knoevenagel condensate derivatives (**9**–**11**) were also derived from curcumin and tested for in vitro antimalarial activity by Mishra et al. [38,67]. The curcumin derivatives were then extendedly synthesized by Dohutia et al. (Table 3) (Figure 14) through further structural modifications producing compounds **14**–**42**, whose reactive methylene α-hydrogen was substituted with a phenyl group, as shown in Figure 15 [37]. It was suggested that the Knoevenagel condensate derivatives were able to inhibit NF-κβ [38,68] and *Pf*ATP6 [39,69] based on in silico studies, and exhibited varying levels of antimalarial and anti-inflammatory activities (Figure 15).

Parameters based on Lipinski’s rule of five, such as solubility and log P, as well as the number of H-bond donors and acceptors, were evaluated in the study. These parameters provide information on the stability and bioavailability of curcumin derivatives and facilitate the design of the most potent derivative structures [71,72]. The Knoevenagel condensate derivatives produced generally exhibited enhanced antimalarial potential, based on their better solubility, binding energy, and percentage of schizont inhibition.

The addition of the phenyl group substituting the α-H (**9**) provides a steric effect and deactivates the reactive methylene site. The extended substituent at the α-carbon position also enables additional interactions with proteins through hydrophobic interactions, resulting in higher free binding energy and percentage of schizont inhibition. The enhanced solubility and log P value of the derivatives translate to their enhanced stability within the hydrophilic and hydrophobic regions of the binding sites on the target protein compared to the parent curcumin (**1**).

The functional groups introduced to the aromatic phenyl also affect the binding and percentage of schizont inhibition of the derivatives. An additional hydroxy group at the meta position (**15**) increases the binding energy through the introduction of additional H-bond donors and acceptors. The hydroxy group also limits the free rotation of the phenyl group, contributing to steric effect and improved interaction with protein, leading to higher free binding energy and percentage of schizont inhibition than derivative (**9**) [73].

**Figure 16:**
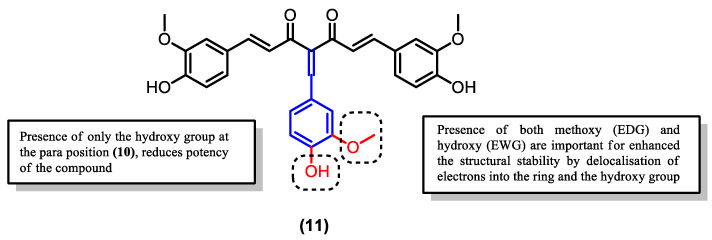
Structure of curcumin derivative (**11**) with both EDG and EWG groups attached to the additional phenyl.

Interestingly, the substitution of the meta hydroxy with a methoxy group combined with the addition of another hydroxy at the para position (**11**) further enhanced the potency of the compound compared to both curcumin (**1**) and derivative (**9**) (Table 2). However, eliminating the methoxy group at the meta position, leaving a monosubstituted para-hydroxy phenol group (**10**), had the opposite effect of reducing its potency to a level lower than even the parent curcumin (Table 2). Thus, the presence of both the electron-donating methoxy group at the meta position and the electron-withdrawing hydroxy group is crucial for the improved antimalarial and anti-inflammatory activities of the curcumin derivative (Figure 16).

**Figure 17:**
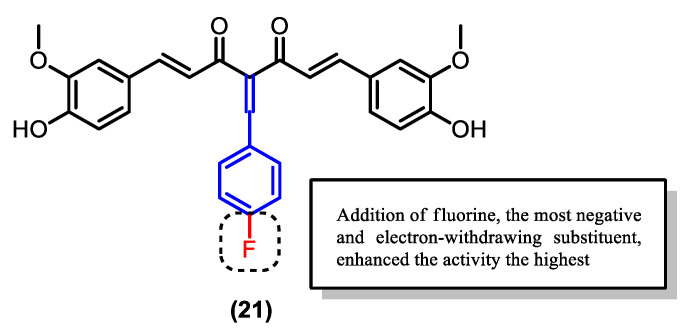
Curcumin derivative (**21**) had the highest potency among the synthesized Knoevenagel condensate derivatives.

Comparing the Knoevenagel condensate derivatives, compound (**21**), which has fluorine (EWG) attached to the phenyl (Figure 17), exhibited the highest percentage of schizont inhibition and the strongest binding. The highly negative and electron-withdrawing fluorine confers structural stability through an inductive effect, similar to the hydroxyl group.

**Figure 18:**
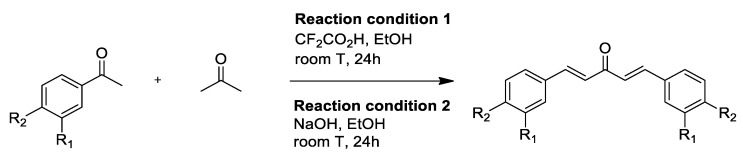
Reaction scheme showing the synthesis of monocarbonyl curcumin derivatives (**43**–**47**).

Another approach to eliminating the keto–enol and the active methylene moieties is through the reduction of the dicarbonyl group into a monocarbonyl group. The compounds reported by Yusuf et al. [74] were not synthesized by modification of the parent curcumin, but instead by total synthesis, as shown in Figure 18. The bioactivity of each monocarbonyl derivative against *P. falciparum* was assessed based on an in silico study of their interaction with the *Pf*DXR protein, as well as an in vitro antimalarial assay, initially developed by Hanne et al. and Mukhtar et al. [74,75,76]. Notably, the activity of the monocarbonyl derivatives was observed to be highly dependent on the substituents attached to the aromatic phenyl rings [74]. 

The mechanism of action for this range of curcumin derivatives was identified to be through the inhibition of the parasite protein *Pf*DXR, which is a proven target for the antimalarial drug fosmidomycin [77]. The carbonyl group at the center of the compounds facilitates a ligand–protein interaction through the formation of two hydrogen bonds at the center of the binding pocket. The hydrophobic aromatic region of the derivatives also contributes to additional hydrophobic interactions, which were not present with fosmidomycin.

**Figure 19:**
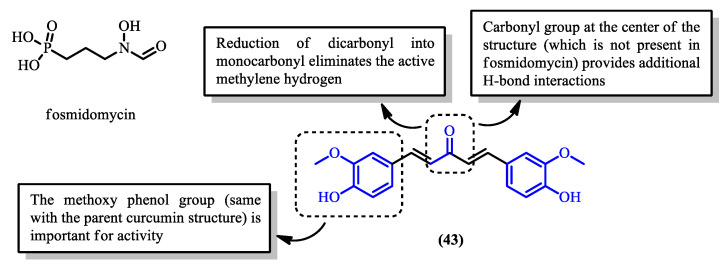
Targeted structural modification sites of monocarbonyl curcumin derivatives.

Monocarbonyl derivative (**43**), which maintained the methoxy phenol group present in the parent curcumin (**1**) (Figure 19), showed 55% parasite elimination in the study. Upon further modification by substituting the methoxy and hydroxy groups into a monosubstituted ring, derivatives (**44**, **45**) were produced. However, the percentage of parasite elimination decreased, indicating their reduced potency. This result supports the importance of the unsubstituted methoxy phenol group of curcumin, as modifying this site leads to the loss of its antimalarial activity [38,39].

## 4. Mechanism of Action of Curcumin

Finding a safe and effective anti-inflammatory therapy remains an obstacle in antimalarial drug development. Malarial infection affects the regulation of the immune system and inflammation levels within the body. Curcumin and its derivatives have emerged as an attractive anti-inflammatory agent due to their wide range of effective cellular-level actions, including regulating the levels of transcription factors [78], cytokine expression [78], and enzymes involved in the progression of infected cells [40,61]. 

### 4.1. Host Proteins as Molecular Targets of Curcumin

**Figure 20:**
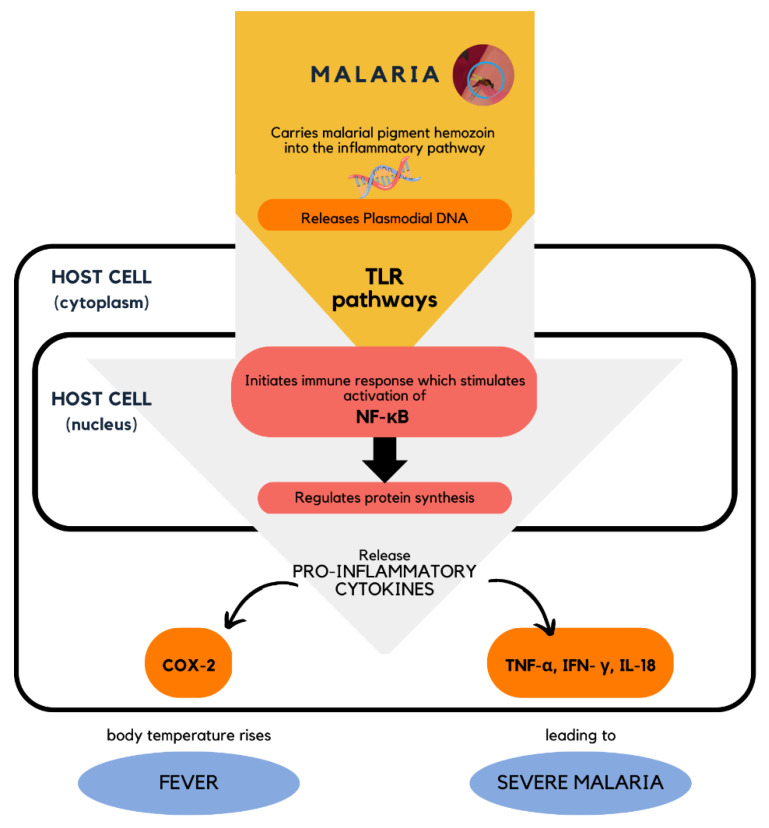
Cellular level mechanism of action of malarial infection in the host cell involving the activation of NF-κB [79].

Since malaria causes dysregulation in the inflammatory response, the elucidation of the mechanisms of action of malarial infection is important in order to understand the pathways and binding targets for inhibition. One potential mechanism involved in regulating the pathophysiology of malaria was identified to involve the Toll-like receptor (TLR) signaling pathways. TLRs, which are located on the cell surface, recognize the released *Plasmodium* DNA and trigger the initiation of immune responses through the activation of NF-κB [79,80]. The transcription factor NF-κB is a critical signaling protein involved in various inflammatory responses and gene expressions [68,81,82]. NF-κB is found in a dormant state in the cytoplasm and will only be transcribed when it is activated and translocated into the nucleus [80,83]. This transcription factor is the main target protein that directly regulates the pro- and anti-inflammatory cytokines within the body, including COX-2, TNF, IL-1, IL-6, IL-8, IL-10, and chemokines [79,84,85,86]. The dysregulation of these cytokines and chemokines will lead to the expression of malarial symptoms such as fever, and if left untreated, will lead to severe malaria (Figure 20). Therefore, NF-κB has become the most targeted factor in the development of antimalarial agents. Another study also established and reported that *P. falciparum* infection leads to the elevation of TNF production, which is the main malarial pathogenic factor, and, hence, could lead to a high risk of severe malaria and even death [87,88,89].

**Figure 21:**
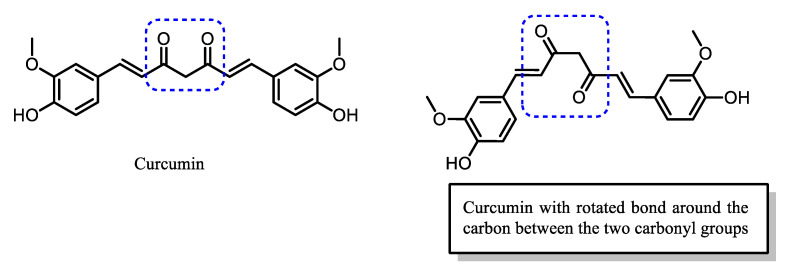
The structural flexibility of curcumin allows for bond rotation around the α-carbon, bridging the two carbonyl groups.

Curcumin has been proven to suppress the activation and translocation of NF-κB into the nucleus, thus, controlling the level of inflammatory cytokines and the larger inflammatory response. Its ability to interact and inhibit various proteins helps in the elucidation and modulation of the pathophysiology of diseases at the molecular level, including malaria [8,90,91]. The interaction of curcumin with proteins is facilitated by its structural flexibility conferred by the presence of the unsaturated diketo group at the center of curcumin [8,85] (Figure 21).

A recent study by Ali et al. on TLR pathways involving the control of the protein kinases Akt and glycogen synthase kinase-3β (GSK3β) also proved NF-κB as a downstream target that regulates anti-inflammatory cytokines [92]. Based on in vivo studies, curcumin was demonstrated to directly inhibit the host GSK3β, leading to the phosphorylation of NF-κB, hence, modulating the regulation of pro- (TNF-α, IFN-γ, and IL-18) and anti-inflammatory (IL-4 and IL-10) cytokine levels [92]. The immunomodulating effect of curcumin in reducing pro-inflammatory cytokine expression can potentially prevent severe and cerebral malaria [93,94]. As evidence to this claim, several in vitro studies have shown that curcumin downregulates pro-inflammatory cytokine production and the expression of cell adhesion molecules in TNF-activated human endothelial cells observed at the trophozoites stage of *P. falciparum* transmission [93]. Furthermore, the inhibition of NF-κB by curcumin also suppresses the generation of reactive oxygen species (ROS), which attenuates the inflammatory response [95,96,97].

### 4.2. Parasite Proteins as Molecular Targets of Curcumin

With regard to parasite proteins, curcumin induces ROS generation in parasite cells, which affects the function of the *Pf*GCN5 histone acetyltransferases (HATs) p300/CREB-binding protein (CBP), thus, inhibiting histone acetylation and the transcription process in the parasite [98]. The generation of ROS is an important antimalarial mechanism as it induces protein and DNA damage within parasite cells, leading to their death [92,99,100]. The specific inhibition of parasite HAT by curcumin prevents the acetylation of K9 and K14 of histone H3. Cui et al. also reported that the antiplasmodial activity of curcumin is attributed, at least in part, to the production of ROS and the downregulation of *Pf*GCN5 HAT activity [48]. 

Another mechanism of action of curcumin is by disrupting the transmission and development of *Plasmodium* parasites at the erythrocytic stage. As erythrocytes burst to release more merozoites, heme is also released. However, the released heme is highly toxic to the merozoites. Thus, the parasites will be stimulated to convert hematin into its detoxified polymeric form, hemozoin [3,47,101]. Curcumin treatment was reported to inhibit the formation of hemozoins in vitro, as observed through transmission electron microscopy in a study using the *P. falciparum* 3D7 strain [44]. This proved that the antimalarial activity of curcumin is similar to that of quinine and chloroquine [101]. 

Uncontrolled parasite transmission in the body can develop into fatal severe anemia or cerebral malaria, whose pathophysiology involves the inflammatory response [91,102]. The excessive stimulation of pro-inflammatory cytokines by the parasite subsequently leads to the sequestration of parasites in the brain [103]. Several reports have indicated the ability of curcumin to eliminate parasites at the trophozoite stage, synergistically and effectively better than artemisinin [37,38,63,104]. This observation was also proven using chloroquine-sensitive (CQS) and chloroquine-resistant (CQR) *P. falciparum* strains, with a proposed mechanism of curcumin action against the parasite proteins *Pf*RIO2-kinase and *Pf*GCN5 HAT [38,104,105].

The Knoevenagel condensate curcumin derivatives mentioned earlier were suggested to target the *Pf*ATP6 parasite protein to explain their schizont inhibition activity. This was based on the established target of the reference antimalarial drug, artemisinin, which shares the same binding pocket on *Pf*ATP6 as curcumin [37,69]. The study applied PreADMET predictions, which demonstrated the attachment and interaction of curcumin and its derivatives with the active site of *Pf*ATP6.

**Figure 22:**
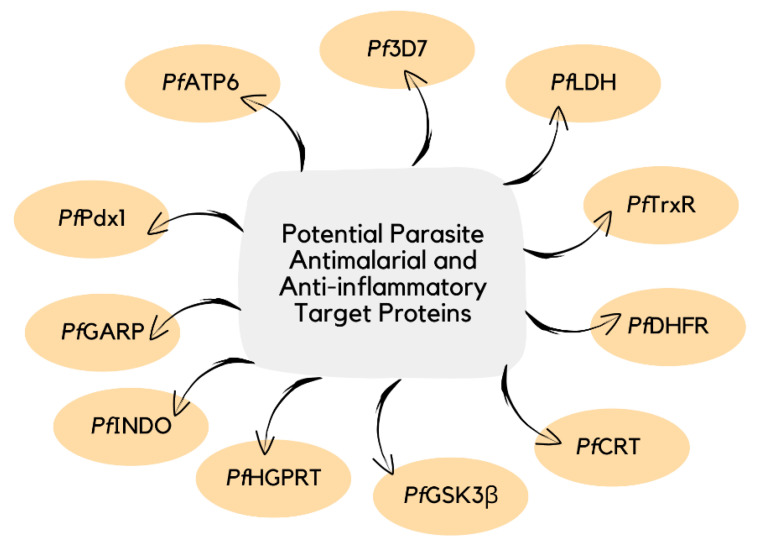
Examples of potential parasite target proteins involved in the pathophysiology of malaria.

The identification of therapeutic targets involved in malarial infection can provide a better understanding of the inhibitory mechanism of antimalarial agents (Figure 22) [39,106]. The current knowledge on the action of curcumin derivatives is limited, as their host-targeting mechanisms have not been entirely established (Table 4) [41,90,107]. Therefore, future research that can explain an in-depth understanding of the molecular-level mechanism of action of curcumin and its bioactive derivatives will not only help in the development of potent antimalarial and anti-inflammatory agents [108] but also for other diseases [109,110,111].

## 5. Conclusions

Various therapeutic effects of curcumin against a wide range of diseases have been extensively reported. Within the context of malarial infection, curcumin exhibits the dual antiplasmodial activity against parasites as well as anti-inflammatory action within the host. However, due to its limited bioavailability, the synthesis of derivatives with better pharmacological profiles than the parent curcumin through structural modifications has been explored. The incorporation of pharmacokinetic property prediction and structure–activity relationship data to rationalize the design of curcumin derivatives with enhanced bioactivity paves the way for the development of more potent antimalarial medicine. While preserving the methoxy phenol groups, which are crucial for the antimalarial and anti-inflammatory activities of curcumin, the reactive methylene group bridging the two carbonyl groups has been targeted for structural modification to generate promising curcumin derivatives. The presence of electron-withdrawing and electron-donating groups within the curcumin derivatives significantly affects their potency, pharmacokinetics, and physicochemical properties, as evaluated in terms of free binding energy, solubility, lipophilicity, IC_50_ values, and percentage of schizont inhibition. Even though the pharmacological effects of curcumin have been attributed to the inhibition of multiple signaling pathways and enzymes in various biological systems, the detailed and specific molecular mechanism underlying its parasiticidal and anti-inflammatory activities remains to be elucidated in detail. The identification of specific target proteins for inhibition that correlates with the mechanism of action of curcumin and its various derivatives is essential for developing safer and more effective therapies against malaria.

## Figures and Tables

**Table 1:** Physicochemical and pharmacokinetic properties of each compound shown in Figure 7, as generated by the Swiss ADME server [62].

Compound	Physicochemical Properties					Lipophilicity	Water Solubility	Drug-Likeness	Pharmacokinetics	
	Molecular Weight (g/mol)	Heavy Atoms	Rotatable Bonds	H-Bond Acceptors	H-Bond Donors	Log P	Log S	Lipinski’s Rule Violation	GI Absorption	BBB Permeability
**1**	368.38	27	8	6	2	3.27	−3.94	No	High	No
**2**	365.38	27	6	6	2	3.60	−4.33	No	High	No
**3**	366.41	27	6	5	3	3.47	−4.33	No	High	No
**4**	460.65	33	9	5	3	4.45	−4.95	No	High	Yes
**5**	478.64	34	9	6	3	4.53	−5.12	No	High	Yes
**6**	505.65	36	10	7	3	3.79	−5.05	1	High	No
**7**	529.54	35	9	5	3	4.82	−6.16	1	High	No
**8**	490.68	35	10	6	3	4.72	−5.05	No	High	No
**9**	456.49	34	9	6	2	3.61	−5.83	No	High	No
**10**	472.49	35	9	7	3	3.55	−5.69	No	High	No
**11**	502.51	37	10	8	3	4.34	−5.77	1	Low	No
**12**	452.45	33	12	8	0	3.83	−4.28	No	High	No
**13**	396.43	29	10	6	0	3.59	−4.37	No	High	Yes
**14**	472.49	35	9	7	3	3.56	−5.69	No	High	No
**15**	472.49	35	9	7	3	3.37	−5.69	No	High	No
**16**	488.49	36	9	8	4	3.44	−5.56	No	Low	No
**17**	504.48	37	9	9	5	3.63	−5.42	1	Low	No
**18**	486.51	36	10	7	2	4.11	−5.91	No	High	No
**19**	486.51	36	10	7	2	3.95	−5.91	No	High	No
**20**	516.54	38	11	8	2	3.86	−5.99	1	High	No
**21**	474.48	35	9	7	2	3.76	−5.99	No	High	No
**22**	492.47	36	9	8	2	3.65	−6.15	No	High	No
**23**	470.51	35	9	6	2	3.62	−6.13	No	High	No
**24**	484.54	36	9	6	2	3.90	−6.44	No	High	No
**25**	484.54	36	9	6	2	3.97	−6.44	No	High	No
**26**	490.93	35	9	6	2	3.82	−6.42	No	High	No
**27**	525.38	36	9	6	2	3.48	−7.02	1	High	No
**28**	501.48	37	10	8	2	2.99	−5.89	1	Low	No
**29**	499.55	37	10	6	2	4.22	−6.07	No	High	No
**30**	501.48	37	10	8	2	2.76	−5.89	1	Low	No
**31**	498.57	37	10	6	2	3.89	−6.69	No	High	No
**32**	500.54	37	9	7	3	3.67	−6.30	1	Low	No
**33**	498.57	37	9	6	2	3.61	−6.74	No	High	No
**34**	500.54	37	11	7	2	4.50	−6.14	1	High	No
**35**	498.57	37	9	6	2	3.60	−6.74	No	High	No
**36**	484.54	36	10	6	2	4.19	−6.41	No	High	No
**37**	488.50	36	9	7	2	3.44	−6.29	No	High	No
**38**	488.50	36	9	7	2	3.86	−6.29	No	High	No
**39**	481.50	36	9	7	2	3.36	−5.78	No	High	No
**40**	471.50	35	9	6	3	3.38	−5.47	No	High	No
**41**	546.48	40	11	10	2	2.14	−5.97	2	Low	No
**42**	502.58	36	10	6	2	4.66	−6.35	1	Low	No
**43**	326.34	24	6	5	2	3.10	−3.95	No	High	Yes
**44**	266.29	20	4	3	2	2.17	−3.93	No	High	Yes
**45**	324.29	24	6	5	0	2.18	−4.22	No	High	No
**46**	294.34	22	6	3	0	3.46	−4.39	No	High	Yes
**47**	392.08	20	4	1	0	3.61	−6.11	1	High	Yes

GI—gastrointestinal; BBB—blood–brain barrier.

**Table 2:** Data reported by Mishra et al. [38] showing the in vitro efficacy of curcumin and its derivatives against *P. falciparum*.

Compound	*P. falciparum* In Vitro Analysis	
	Chloroquine-Sensitive FCK2	Chloroquine-Resistant MP-14
	IC_50_ (μM)	IC_50_ (μM)
**1**	3.25	4.21
**2**	8.44	7.92
**3**	0.48	0.45
**4**	8.48	9.10
**5**	2.42	2.10
**6**	0.87	0.89
**7**	4.65	4.80
**8**	22.60	24.56
**9**	3.89	4.12
**10**	5.85	5.36
**11**	0.92	0.75
**12**	2.34	2.51
**13**	7.86	8.40

**Table 3:** In vitro and in silico assessment of antimalarial activity of curcumin derivatives by Dohutia et al. [37].

Compound	MW (g/mol)	logP	H-Bond Donor	H-Bond Acceptor	Solubility (mg/L)	IC_50_ (μM)	Free Binding Energy (kcal/mol)	% Schizont Inhibition	
								5 μg/mL	50 μg/mL
(**21**)	474.48	5.43	2	6	1382.51	-	−6.75	97.8	100
(**15**)	472.49	4.97	3	7	1869.61	-	−5.89	89.5	100
(**9**)	456.49	5.33	2	6	1583.74	3.89	−5.35	80.1	100
Curcumin	368.38	3.29	2	6	7.475	3.25	−5.25	79.6	100
(**10**)	472.49	4.97	3	7	1869.61	5.85	−3.87	-	-

**Table 4:** Antimalarial and anti-inflammatory activities of curcumin and its derivatives.

Activity	In Vitro/In Vivo*/*In Silico Evidence	References
Antiplasmodium *Pf*ATP6	Curcumin (**1**) reduced *P. falciparum* viability, causing parasitic cell proliferation to decrease. - Reported IC_50_ value: 5 μM.	[69]
	Curcumin (**1**) and its derivatives (**9**, **14**, **15**, **19**, **21**, **23**, **27**, and **28**) showed 100% inhibition of *P. falciparum* growth upon a 50 μg/mL dose of treatment.	[37]
	Molecular docking results validated binding of curcumin (**1**) and its derivatives to *Pf*ATP with favorable free binding energy. - Reported free binding energy: Curcumin derivative (**21**): –6.75 kcal/mol (higher than both artemisinin (–6.73 kcal/mol) and curcumin (–5.25 kcal/mol), hence, better interaction with the protein). - Hydrogen bonding with Lys1213 and Leu1044	[37]
	In vitro study using CQR *P. falciparum* showed potent antimalarial activity of curcumin (**1**), with reported IC_50_ value of ~5 μM. In vivo treatment of *P. berghei*-infected mice with 100 mg/kg curcumin showed: - More than 80% inhibition of parasitic growth. - A 29% increase in survival rate.	[23,25]
	Curcumin treatment on *P. berghei*-infected C57BI/6 mice delayed mice death by 10 days and prevented cerebral malaria. Dose: 50 mg/kg, twice daily for 6 days.	[112]
	Curcumin exhibited antimalarial activity in *P. berghei*-infected mice. Dose: 300 mg/kg daily for 4 days (60.22% parasitemia inhibition). Dose: 80 mg/kg daily for 4 days (60.21% chemosuppressive effect).	[113,114]
Antiplasmodium *Pf*3D7	Curcumin (**1**) showed potential inhibition of parasite transmission at the trophozoite stage. Curcumin derivative (monocarbonyl curcumin) - Reported IC_50_ values against CQS: 1.97 μM, CQR: 1.69 μM. Curcumin derivative (**13**) - Reported IC_50_ value: 1.97 μM.	[115]
Antiplasmodium *Pf*DXR	In silico and in vitro studies validated synergistic binding of curcumin (1) to *Pf*DXR protein with fosmidomycin. - Presence of methoxy substituent on the phenyl groups facilitated parasite elimination: (**43**)—55%. (**46**)—57%.	[74]
Antiplasmodium *P*GCN5 HAT	In vitro study suggested curcumin (**1**) as a potent inhibitor of p300/CBP (CREB-binding protein) as tested on four *P. falciparum* strains. - Reported IC_50_ values—3D7: 24.69 μM, D10: 22.93 μM, 7G8: 29.61 μM, Dd2: 27.45 μM.	[92]
Antiplasmodium *Pf*TrxR	In vitro study using CQS (D6 clone) and CQR (W2 clone) *P. falciparum* strains showed that curcumin (**1**) inhibited *Pf*TrxR protein with an IC_50_ value of 2 μM.	[116]
Antiplasmodium *Pf*HGPRT *Pf*SAHH	In silico simulation using Molegro Virtual Docker (MVD) and admetSAR showed high binding energy of curcumin (**1**) to the protein. - Reported binding energy: - *Pf*HGPRT: −175.97 kcal/mol. - *Pf*SAHH: −138.30 kcal/mol.	[107,117]
Antimalaria ROS	In vitro study showed that curcumin (**1**) induced intracellular ROS production related to PPARɣ/Nrf2 activation. - Reported IC_50_ value: 10 μM.	[95,118]
Antimalaria	In vitro study using NF54 intraerythrocytic-form *P. falciparum* strain reported highly potent antiparasitic activity of curcumin (**1**). - Reported IC_50_ value: 0.59 μM.	[119]
	In vitro study using 3D7 clone strain of *P. falciparum* reported synergistic antimalarial effect of curcumin (**1**) with dihydroartemisinin and reduction in hemozoin formation upon several consecutive treatments of curcumin. - Reported IC_50_ value: 2.2 μg/mL.	[100]
	In vitro study showed the effectiveness of curcumin–artemisinin combination therapy with additive interaction in killing *P. falciparum*. In vivo study using *P. berghei*-infected mice showed 100% survival upon treatment. Dose: 750 µg.	[119]
	In vivo study on *P. berghei* ANKA-infected mice revealed treatment of curcumin (**1**) reduced parasitemia level and increased survival rate. Dose: 50 mg/kg daily.	[120]
	In vitro study shows reported IC_50_: - Curcumin Encapsulated to PLGA: 292.6 µg/mL. - Curcumin (1): 1000 µg/mL. In vivo study on *P. berghei*-infected mice and murine RAW 264.7 macrophages using curcumin encapsulated in PLGA showed 56.8% parasite suppression (higher than free curcumin with 40.5% suppression). Dose: 5 and 10 mg/kg.	[121]
Anti-inflammatory COX-2	In vitro study using DPPH radical-scavenging assay showed anti-inflammatory activity of curcumin and its derivatives. Reported IC_50_ value and % inhibition: - Curcumin (**1**)—IC_50_ value: 11.06 μM, 35.0% inhibition. - Derivative (**2**)—IC_50_ value: 10.71 μM, 58.1% inhibition. - Derivative (**3**)—IC_50_ value: 9.70 μM, 61.0% inhibition.	[65]
	Molecular docking using FlexX program validated COX-2 as a target protein and showed binding of curcumin (**1**) and curcumin derivatives (**2**, **3**). Favorable interactions: - Hydrogen bonding interaction with Arg120. - van der Waals interactions with Val523, Val116, Ala516, and Try355.	[65]
Anti-inflammatory NF-κB	In vivo study on *P. berghei* ANKA-infected mice upon treatment of curcumin (**1**) showed inhibition of NF-κB activation, which reduced expression of adhesion molecules and suppressed pro-inflammatory cytokines level. Dose: 100 mg/kg daily for 4 days.	[122]
Anti-inflammatory GSK3β	In vivo study on *P. berghei* NK65-infected rats upon treatment of curcumin (**1**) showed inhibition of host GSK3β, leading to the phosphorylation of NF-κB and regulation of pro- (decrease in serum TNF-α and IFN-γ levels) and anti-inflammatory (IL-10 and IL-4) cytokines. Dose: 3, 10, and 30 mg/kg.	[88]
Anti-inflammatory	In vivo study on *P. berghei* NK65- and ANKA-infected mice upon treatment of curcumin (**1**). Reported activity: - Significant decrease in inflammatory cytokine levels including serum p53, TNF-α, CRP, and IL-6. - Inhibition of mPT pore opening, F_0_F_1_ ATPase activity and mLPO. Dose: 50 mg/kg.	[123]

## Data Availability

Data sharing not applicable.
